# Lethal and sublethal effects of synthetic and bio-insecticides on *Trichogramma brassicae* parasitizing *Tuta absoluta*

**DOI:** 10.1371/journal.pone.0243334

**Published:** 2021-07-30

**Authors:** Zahra Nozad-Bonab, Mir Jalil Hejazi, Shahzad Iranipour, Mehdi Arzanlou, Antonio Biondi

**Affiliations:** 1 University of Tabriz, Tabriz, Iran; 2 Department of Plant Protection, Faculty of Agriculture, University of Tabriz, Tabriz, Iran; 3 Department of Agriculture, Food and Environment, University of Catania, Catania, Italy; Institut Sophia Agrobiotech, FRANCE

## Abstract

The tomato leaf miner (TLM), *Tuta absoluta* (Meyrick), is an invasive tomato pest found worldwide. Sustainable control strategies aimed at increasing biological control approaches and decreasing chemical inputs are required, due to the tendency to develop insecticide resistance. In this study, the lethal and sublethal effects of four chemical insecticides (abamectin, indoxacarb, chlorantraniliprole, and spinosad) and the sublethal effects of the entomopathogenic fungus *Metarhizium anisopliae* (Metschnikoff) on a widespread TLM egg parasitoid, *Trichogramma brassicae* Bezdenko, were estimated. Concentration mortality response bioassays enabled the estimation of lethal concentrations of the tested insecticides for the parasitoids, with chlorantraniliprole having the lowest LC_50_ and indoxacarb the highest. The LC_25_ and LC_50_ of the tested insecticides on the TLM were sprayed on eggs and then offered at three time intervals to the parasitoids. The fertility and other life table parameters of the individuals emerging from the treated eggs were estimated. All of the chemical insecticides, but not the fungus, had harmful effects on *T*. *brassicae*. The insecticide applications caused a 3.84–5.17 times reduction in the net reproductive rate (*R*_*0*_) compared with the control. No parameters were affected by spraying the fungus in the 0h treatment, but effects were recorded at 24 and/or 48h, except for the gross reproduction rate (*GRR*). The value of the intrinsic rate of increase (*r*_*m*_) also decreased to 0.528–0.617 after the insecticide treatments. The doubling time (*DT*) increased in all treatments compared to the control. Nevertheless, the generation time (*T*) was only very slightly affected. In addition, in the combination experiments, *M*. *anisopliae* showed a remarkable synergism with *T*. *brassicae* in controlling TLM eggs. These results indicate that low levels of lethal effects on key biological control agents should be considered in the choice of insecticides to be included in sustainable TLM control packages.

## Introduction

The tomato leaf miner (TLM), *Tuta absoluta* (Meyrick) (Lep: Gelechiidae(, is a major tomato pest worldwide [1]. Native to South America, this pest has spread rapidly through most continents over the past 15 years. It was first reported outside of Latin America in Spain in 2006 [2]. TLM was then identified in most European, African and Asian countries, including Middle Eastern countries, causing a 50–100% loss of tomato crops [3]. The comprehensive review of Han et al. [4–6] suggests that this pest, due to its ability to adapt to newly invaded areas, high reproduction potential and other traits, can be very harmful for tomatoes in Middle Eastern countries such as Iran. It was first report in Turkey in 2009, and invaded Iran’s fields in 2009, possibly via its borders with Turkey or Iraq [7].

The females can lay up to 260 eggs and the larvae are leaf miners that feed on the leaf mesophyll of tomato and other solanaceous plants, between the two epidermises [8, 9]. This feeding behavior, the short development time, the production of several generations and its rapid adaptation to different ecological conditions, means that this insect has been an extremely problematic and serious pest for tomatoes, and can cause 100% damage if no suitable management measures are adopted [1].

Several studies have attempted to estimate the effectiveness of synthetic or bio insecticides on TLM [10–16]. However, insecticide application causes undesirable effects, such as the development of resistance and damage to non-target organisms, including insect natural enemies [17–21]. The *slower acting* modern insecticides can cause various sublethal effects that finally lead to pest suppression and limit the evolution of resistance [22]. However, TLM has been shown to be resistant to various chemicals, including new insecticides [23]. Reyes et al. [14] showed that increasing the activity of the detoxifying enzymes at the larval stage caused resistance to spinosad. Silva et al. [24] showed that a single nucleotide change may be behind the resistance of *T*. *absoluta* to spinosad. Evidence for TLM resistance to abamectin, indoxacarb and chlorantraniliprole has also been found in newly invaded areas [13, 14].

The evolution of resistance to insecticides can cause deleterious effects on various life table parameters, and such fitness costs may have direct implications for pest management [25–29]. Nevertheless, low lethal doses of some insecticides can increase resistance and the fitness of pest progeny, thus leading to the resurgence of the pest [30]. Assessing which insecticides are effective and monitoring the evolution of their resistance to these compounds, or the deleterious effects on biocontrol agents, is necessary when identifying a suitable and effective IPM program. In this context, the implementation of the biological control services provided by parasitoids, predators and entomopathogens is a sustainable strategy for many crops [31–37]. The biological enemies of TLM such as parasitoids, predators and pathogens that have been reported as persistent, and the many factors of the biological agents that act on this pest, such as food, shelter, and genetic improvement, have been extensively studied [33, 34, 38–43].

Among the various parasitoid species parasitizing TLM in both the native and the newly invading range [1, 44, 45], the idiobiont oligophagous species *Trichogramma* has been shown to have a good potential for use in integrated pest management (IPM) programs [46–50]. These parasitoids have been used in over 30 countries to control almost 20 different crop pests [51]. Ahmadipour et al. [52] studied six populations of *Trichogramma* on TLM eggs in laboratory conditions in Iran. They reported significant differences between the parasitoid populations, with the highest parasitism rate being 54%.

The integration of *Trichogramma* spp. with other control measures such as predators, entomopathogens and pheromone traps to control TLM have been considered in the context of Iran, and studies show that these egg parasitoids are extremely important in the IPM programs of TLM [53–55]. The wide range of tolerance to environmental changes, the ability for mass rearing, the capacity to kill their hosts before damage occurs and a measurable host preference despite a wide host range are advantages that make *Trichogramma* spp. a valuable biological control agent [46, 51]. The host species may dramatically affect the fitness of *Trichogramma* parasitoids, in terms of their size, longevity, fecundity and host acceptance [53, 56–58]. Some Trichogrammatidae species, such as *Trichogramma achaeae* (Nagaraja & Nagarkatti) [48, 59], *T*. *euproctidis* (Girault) [49, 60], and *T*. *evanescens* (Westwood) [61], have been reported as effective egg parasitoids of TLM. Unfortunately, none of these species are extensively used commercially. *Trichogramma brassicae* Bezdenko (Hym.: Trichogrammatidae) is the only species in Iran that is reared, albeit to a limited extent, and thus has potential for future application in Integrated Pest Management (IPM) programs in Iran.

Pathogens are also important biocontrol agents of TLM that are currently used commercially. Of these, fungi are promising due to their diversity and adaptation to agroecosystems [62]. Most of the commercially used fungal entomopathogens belong to the genera of *Metarhizium*, *Beauveria*, *Lecanicillium* and *Isaria*. They can directly penetrate into the insect cuticle and are able to cause epizootics. They can also be easily produced in large quantities [63, 64]. *Metarhizium anisopliae* (Metschnikoff) Sorokin (Hypocreales: Clavicipitaceae) is a well-known entomopathogen that belongs to Pezizomycotina: Sordariomycetes [65]. A high virulence of this fungus has been reported on TLM eggs, larvae, pupae and adults [32, 36, 66]. *Metarhizium anisopliae* is a promising entomopathogenic fungus that can be used at a commercial level [67]. They should therefore be properly integrated with other control tools to provide effective and sustainable control.

Spinosad is a bioinsecticide used in traditional and organic cultures, and thus examining the side effects of spinosad on pest or biological control agents is very important. Biondi et al. [68] found that spinosad is more toxic to Hymenoptera than other parasitoid orders, although it is more selective in terms of bees. As with most modern bioinsecticides, spinosad can cause several sublethal rather than lethal effects. Thus, a comprehensive evaluation of insecticide effects should be conducted and the sublethal effects on natural enemies should also be considered [17, 46, 69–71]. Therefore, combining insecticides and parasitoids may not have the expected results. Integrating chemical and biological control measures is crucial in pest management programs. An effective combination requires an awareness of the side-effects of insecticides on other components of the environment, and initially on key natural enemies. We selected the most effective insecticides against *T*. *absoluta* based on a previous study [72].

As the compounds used against TLM directly and/or indirectly affect its natural enemies, evaluating such effects is necessary when choosing the suitable insecticides to be included in TLM control programs. In most studies, a female-cohort life table has been used to evaluate insecticide effects on an insect population [73], but recently the male cohort was included by Huang and Chi [74]. The age-stage two-sex life table evaluates population growth parameters based on both sexes and stage differentiation, which removes the inherent errors of female-based life tables. Additionally, this method simplifies the data recording method and saves time and labor [75].

Using this framework, we conducted this study to evaluate the lethal and sublethal effects of insecticides (abamectin, indoxacarb, spinosad and chlorantraniliprole) and the sublethal effects of entomopathogen *M*. *anisopliae* (broadly used against TLM) on *T*. *brassicae*, which is the prevalent egg parasitoid species of *Trichogramma* in Iran.

## Materials and methods

### Rearing *Tuta absoluta*

Different larval instars of *T*. *absoluta* were collected from a damaged tomato field in Bilasuvar County (39° 39ʹ 31.37ʺN 48° 34ʹ 67.06ʺE) in the Ardabil province of Northwest of Iran and were moved to a greenhouse section of the Department of Plant Protection, University of Tabriz, Tabriz, Iran. The larvae were kept at 27 ± 2°C, 50 ± 10% RH and at a 16: 8 h (L: D) photoperiod and fed on the foliage of greenhouse-grown tomato plants and maintained until adult emergence. The adults were then transferred to 80 × 70 × 60 cm wooden cages covered with organdy cloth, in which two or three potted tomato plants (20–30 cm height) were included, and were allowed to mate and lay their eggs on the plants for 24 hours. The adults were fed with 10% sugar solution (renewed every three days). After 24 h the plants were shaken within the cage to remove the moths from them. Twenty-four-hour old eggs were then used in the bioassays.

### *Trichogramma brassicae* rearing

The egg parasitoid *T*. *brassicae* was provided from a private insectarium in Parsabad, Ardabil province. The stock culture of the parasitoid was reared on *Ephestia kuehniella* (Zeller) (Lep.: Pyralidae) eggs within glass tubes (1 cm diameter, 6 cm length) in a growth chamber at 27±1°C, 50±5% RH and a 16:8 h (L:D) photoperiod. These parasitoids were reared on TLM eggs for two further generations before being used in the experiments.

### *Metarhizium anisopliae* cultures

The entomopathogenic fungus *M*. *anisopliae* was obtained from the laboratory of Biological Control of Insects, University of Tehran. The fungus was cultured on a potato dextrose agar (PDA) medium in Petri dishes (9 cm in diameter) at 25±1°C. Ten days later, the cultures with well-developed spores were washed with distilled water + 0.2% surfactant Tween-80^®^. After filtering the spore suspension using glass wool, the number of spores was counted using a haemocytometer (Assistent^®^).

### Lethal effects of insecticides on *Trichogramma brassicae*

Based on a previous study [72], four chemical insecticides, spinosad (Laser^®^ 48 SC), indoxacarb (Steward^®^ 30 WG), abamectin (Vertimec^®^ 1.8 EC) and chlorantraniliprole (Coragen^®^ 18.5 SC) were chosen as effective insecticides for the *T*. *absoluta* control.

The lethal effects of these insecticides on *T*. *brassicae* were examined. To assess these effects, 120 tomato leaf miner eggs were glued onto a piece of paper (1.5 × 3 cm) in a glass tube (6 cm length, 1cm diameter) and exposed to *T*. *brassicae* females. Five days later, when the blackhead stage of parasitized eggs appeared, they were sprayed by selected insecticides using a Potter spray tower (Burcard Scientific^®^) (5 ml insecticide solution under 0.5 bar pressure). The ranges of concentrations were 0.9–12.6; 0.6–4.5; 0.005–0.11; 0.024–0.72 mg a.i. L^-1^ for abamectin, indoxacarb, chlorantraniliprole and spinosad, respectively. Tween-80^®^ was used as the surfactant at a concentration of 0.05% (v/v) in all treatments. In the control, the eggs were sprayed with distilled water + Tween-80^®^. Five days later the number of emerged parasitoids was recorded and the LC_25_ and LC_50_ values were estimated. The experiment involved three replicates with 40 insects each. The mortalities were corrected using Abbott’s formula [76] and the LC_50_ values were estimated using the probit procedure of SPSS.

### Sublethal effects of insecticides on *Trichogramma brassicae*

For the evaluation of the sublethal effects of the abovementioned insecticides and the entomopathogenic fungus, the LC_25_ values of the chemical insecticides and the LC_50_ value of the entomopathogenic fungus (0.07, 1.82, 1.44 and 0.056 mg ai/l of spinosad, indoxacarb, abamectin and chlorantraniliprole, respectively), and 1.97 × 10^4^ spore/ml of the fungus as obtained in a previous study [72], were sprayed on 50 TLM eggs on a piece of paper (3 cm length, 1.5 cm width). The treated eggs were exposed to the parasitoids 0, 24 and 48h later. Thirty couples of the parasitoids were selected and transferred into glass tubes (6 cm length, 1cm diameter). Very small honey droplets (20%) were placed on a piece of paper (2 cm length, 1 cm width) and deposited in the tubes. All of the tubes were kept in a growth chamber (27 ± 1°C, 60 ± 10% RH and 16: 8 h photoperiod) until the end of the study. The development time, emergence rate, longevity and fecundity of the progeny were thus assessed.

### Combination of *Trichogramma brassicae* and insecticides or entomopathogenic *Metarhizium anisopliae*

In another experiment, 20 TLM eggs were topically treated with LC_25_ of the insecticides or LC_50_ of *M*. *anisopliae* using a Potter spray tower and were then exposed to one *T*. *brassicae* female immediately after drying, 24h or 48h after incubation in laboratory conditions, and then kept in a growth chamber until larvae emergence. This experiment was repeated for 30 parasitoids. The number of *T*. *brassicae* adults and TLM larvae were counted at the end of the experiments and the mortality rate was estimated as the number of dead and parasitized eggs compared to those available. This study involved three sets of control treatments: 1. the untreated eggs on the leaflet, to ensure they were healthy; 2. the parasitized eggs without insecticides, to ensure successful parasitism; and 3. The eggs treated with insecticides and *M*. *anisopliae*, to ensure the effectiveness of the insecticides and the entomopathogen. Each experiment involved four replications.

### Data analysis

The life table parameters include the gross reproductive rate (*GRR*), which is the number of females produced by a single female during her lifetime, the net reproductive rate (*R*_*0*_), which represents the total number of female offspring that an individual female can produce during her lifetime, and the intrinsic rate of increase. In addition, Fecundity (*F*_*x*_) is the physiological maximum potential reproductive output of an individual (*r*_*m*_), which gives the instantaneous rate of population growth of a stable population (a population with constant age-stage distribution) under defined conditions. The finite rate of increase (*λ*) is the per capita rate of increase in females per unit time in a population with a stable age-stage distribution. Generation time (*T*) is the length of time that a population requires to increase to the *R*_*0*_-fold of its initial size at a stable age-stage distribution. Doubling time (*DT*) is the duration of the doubling of the population. The age-stage specific survival rate (*S*_*xj*_) gives the probability that a newly laid egg will survive to age *i* and stage *j*. The age-specific survival rate (*l*_*x*_) simplifies the survival of different development periods as it does not consider differences among individuals. The age-specific fecundity (*m*_*x*_) is the average number of female offspring a female has at age *x*. Age-specific maternity *(l*_*x*_*m*_*x*_) is included along with age-stage specific life expectancy (*E*_*xj*_), which is the expected duration of time that an individuals of age *x* and stage *j* will live. Finally, the age-stage reproductive value (*V*_*xj*_) shows the likelihood of a population from age *x* to stage *j* producing future offspring, which was estimated with the TWOSEX MSChart computer program [77–82]. The standard errors of the life table parameters can be estimated via the bootstrap technique with 100,000 times resampling, and the differences between the population parameters can be compared using the paired bootstrap test based on the confidence intervals of differences, as implemented in the TWO SEX-MSChart software [83].

The risk quotients for the tested insecticides were calculated to determine their safety for *T*. *brassicae* as follows: risk quotient = field recommended dose (mg a.i. ha^-1^)/ LC_50_ for parasitoid (mg a.i. L^-1^). Insecticides with risk quotient values below 50 are considered safe, while those between 50–2500 are slightly to moderately toxic and those higher than 2500 are highly toxic [84, 85]. The method modified from that of Koppenhöfer and Kaya [86] by Yii et al. [87] was used to categorize the binary relationship between different control agents from the antagonistic, additive or synergistic categories. This method is based on the testing discrepancy between the observed mortality and the expected mortality calculated as ME = MC + MB (1 –MC/100) by using a chi-square test (df = 1), where ME, MC and MB are expected mortality, mortality by a chemical factor and mortality by a biological factor, respectively.

In the chi-square test, χ^2^ = (MCB–ME)^2^/ME, where MCB is the observed mortality for the parasitoid–insecticide combinations. If the calculated χ^2^ value exceeds the critical value of the Chi square table (3.84, df = 1), a non-additive (synergistic or antagonistic) relation between the two agents is implied [88], otherwise it is an additive relation. If the null hypothesis of the additive relation is rejected, and the D = MCB–ME has a positive value, then the relation is considered a synergistic type, but is still therefore an antagonistic relation.

## Results

### Lethal effects

The LC_50_ values estimated for the examined insecticides on *T*. *brassicae* are shown in Table 1. The results indicated that chlorantraniliprole had the lowest LC_50_ value (0.02 mg a.i./l), followed by spinosad, abamectin and indoxacarb (0.14, 0.31 and 1.34 mg a.i./l). The LC_50_ values of spinosad, abamectin, indoxacarb and chlorantraniliprole on TLM (0.14, 0.36, 3.99 and 0.11 mg a.i./l, respectively) were estimated by Nozad-Bonab et al. [72]. The LC_50_ value of chlorantraniliprole on *T*. *brassicae* was 5.33 times higher than that of the *T*. *absoluta* examined in our previous study [72]. In addition, Indoxacarb was 2.975 times more toxic for the parasitoid than the tomato leaf miner. Spinosad and abamectin had almost similar toxic effect both on the host and parasitoid. Based on the risk quotient for insecticides, abamectin, spinosad and indoxacarb were classified as slightly to moderately toxic for the parasitoid, with risk quotient values of between 50 and 2500. Chlorantraniliprole was considered highly toxic for the parasitoid (Table 1).

**Table 1:** Summary of probit analysis results and estimated Lethal Concentrations (LC_50_ and LC_90_) of the chemical insecticides tested on *Trichogramma brassicae* juvenile stages within *Tuta absoluta* eggs.

Pesticides	Slope ± SE	LC_25_ (mg a.i./l)	LC_50_ (mg a.i./l)	LC_90_ (mg a.i./l)	Label dose for *T*. *absoluta* (mg a.i./l)	Risk quotient	*χ*^*2*^(*df)*	P-Value
**Spinosad**	**0.99 ± 0.12**	**0.03 (0.02–0.04)**	**0.14 (0.11–0.19)**	**2.81 (1.51–7.38)**	**0.12**	**857.14**	**0.73 (3)**	**0.86**
**Abamectin**	**1.29 ± 0.15**	**0.09 (0.06–0.13)**	**0.31 (0.24–0.38)**	**3.05 (1.92–6.3)**	**0.022**	**69.32**	**0.48 (3)**	**0.92**
**Indoxacarb**	**1.79 ± 0.20**	**0.56 (0.4–0.71)**	**1.34 (1.13–1.56)**	**6.95 (5.09–11.13)**	**0.18**	**134.33**	**0.34 (3)**	**0.95**
**Chlorantraniliprole**	**1.14 ± 0.13**	**0.005 (0.003–0.008)**	**0.02 (0.002–0.03)**	**0.28 (0.17–0.61)**	**0.092**	**4625**	**1.33 (3)**	**0.72**

### Sublethal effects

Longevity in the control was 3.32 days and 1.32, 1.47, 1.54, 1.32 and 3.06 d for the adult parasitoids that emerged from *T*. *absoluta* eggs sprayed with abamectin, chlorantraniliprole, spinosad, indoxacarb and *M*. *anisopliae*, respectively. The chemical insecticides caused significant deleterious effects on all life table parameters compared to the control (P<0.05) (Table 2). However, *M*. *anisopliae* had a moderate and delayed effect on the biostatistics of *T*. *brassicae*. For example, *M*. *anisopliae* had no significant time-dependent effect on *GRR* compared to the control (P<0.001), no matter how long from the exposure time (0, 24 or 48h). Unlike chemical insecticides, the entomopathogenic fungus appeared to have no effects on the intrinsic rate of increase at 0 and 24h after treatment (P<0.05). Low lethal doses of the tested insecticides lengthened the generation time in some treatments and doubled the time in most. This also occurred in some cases of *M*. *anisopliae* (P<0.05). However, no significant interaction was observed between the insecticides and exposure times in most of the experiments. The entomopathogenic fungus *M*. *anisopliae* developed and killed the eggs, and the surviving eggs were of a low quality, which adversely affected the parasitoid preference, but this negative effect was less than that for the insecticides.

**Table 2:** Life table parameters (means ± SE) estimated for *Trichogramma brassicae* developed on *Tuta absoluta* eggs sprayed with a low lethal concentration of 25% at three-time intervals.

	Timing (hrs) of parasitoid release after the treatment	*GRR*±SE	*R*_*0*_±SE	*r*_*m*_±SE	*λ*±SE	*T*±SE	*DT*±SE
***Trichogramma brassicae***		**38.582 ± 4.178**	**31.342 ± 3.933**	**0.303±0.011**	**1.354±0.015** ^**a**^	**11.375±0.082**	**2.289±0.089**
**chlorantraniliprole**	**0**	**13.665 ± 2.25**	**6.948 ± 0.972**	**0.175 ± 0.013**	**1.910 ± 0.016**	**11.098 ± 0.171**	**3.965 ± 0.322**
**chlorantraniliprole**	**24**	**11.908 ± 1.849**	**7.180 ± 1.052**	**0.178 ± 0.013**	**1.194 ± 0.016**	**11.122 ± 0.137**	**3.911 ± 0.317**
**chlorantraniliprole**	**48**	**9.600 ± 1.511**	**6.824 ± 1.011**	**0.176 ± 0.014**	**1.192 ± 0.017**	**10.929 ± 0.126**	**3.946 ± 0.341**
**abamectin**	**0**	**17.411 ± 2.894**	**8.157 ± 1.223**	**0.187 ± 0.014**	**1.205 ± 0.017**	**11.228 ± 0.146**	**3.707 ± 0.298**
**abamectin**	**24**	**14.182 ± 3.24**	**7.378 ± 1.176**	**0.180 ± 0.015**	**1.198 ± 0.018**	**11.074 ± 0.165**	**3.840 ± 0.349**
**abamectin**	**48**	**13.516 ± 2.318**	**7.079 ± 1.090**	**0.178 ± 0.015**	**1.195 ± 0.017**	**11.659 ± 0.154**	**3.885 ± 0.348**
**spinosad**	**0**	**14.724 ± 1.947**	**7.158 ± 0.116**	**0.176 ± 0.015**	**1.193 ± 0.017**	**11.164 ± 0.185**	**3.931 ± 0.358**
**spinosad**	**24**	**9.088 ± 1.616**	**6.363 ± 1.104**	**0.168 ± 0.016**	**1.183 ± 0.019**	**10.994 ± 0.123**	**4.119 ± 0.451**
**spinosad**	**48**	**13.531 ± 2.064**	**7.541 ± 1.183**	**0.181 ± 0.014**	**1.199 ± 0.017**	**11.141 ± 0.193**	**3.822 ± 0.329**
**indoxacarb**	**0**	**13.317 ± 2.202**	**6.243 ± 1.003**	**0.165 ± 0.015**	**1.180 ± 0.018**	**11.064 ± 0.165**	**4.188 ± 0.429**
**indoxacarb**	**24**	**13.308 ± 2.119**	**6.184 ± 0.946**	**0.162 ± 0.014**	**1.176 ± 0.016**	**11.223 ± 0.145**	**4.271 ± 0.401**
**indoxacarb**	**48**	**11.729 ± 1.975**	**6.061 ± 0.917**	**0.160 ± 0.014**	**1.174 ± 0.016**	**11.220 ± 0.132**	**4.317 ± 0.413**
***M*. *anisopliae***	**0**	**41.155 ± 4.647**	**27.679 ± 3.364**	**0.281 ± 0.011**	**1.325 ± 0.015**	**11.808 ± 0.169**	**2.465 ± 0.104**
***M*. *anisopliae***	**24**	**43.902 ± 4.325**	**23.022 ± 2.758**	**0.266 ± 0.011**	**1.305 ± 0.014**	**11.769 ± 0.182**	**2.601 ± 0.108**
***M*. *anisopliae***	**48**	**32.754 ± 3.950**	**21.103 ± 2.410**	**0.262 ± 0.011**	**1.300 ± 0.013**	**11.622 ± 0.149**	**2.642 ± 0.106**

**Fig 1:**
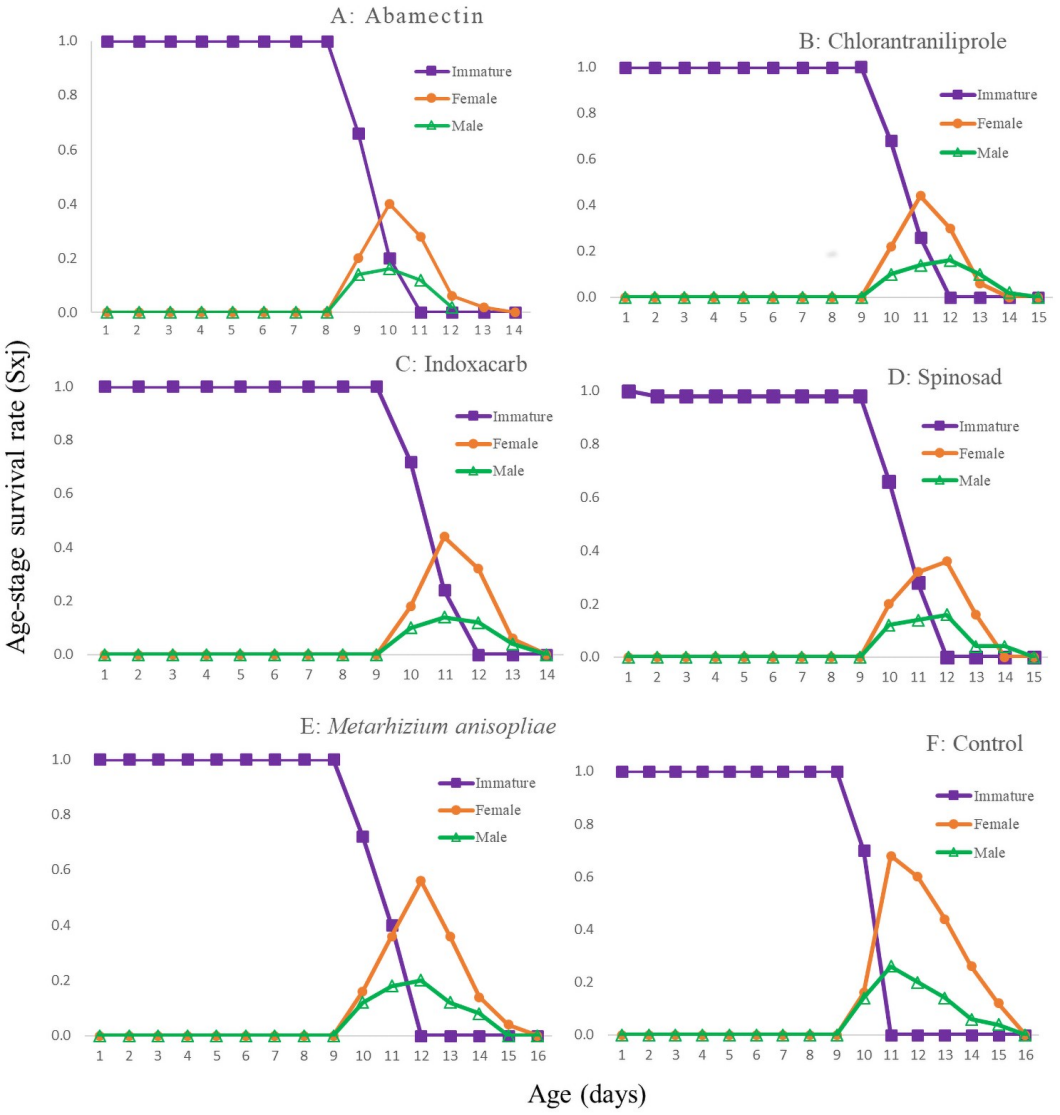
Age-stage specific survival rate (*S*_*xj*_) of the *T*. *brassicae* exposed to different pesticides 48 hours after spraying, A: Abamectin, B: Chlorantraniliprole, C: Indoxacarb, D: Spinosad, E: *M*. *anisopliae*, F: control.

**Fig 2:**
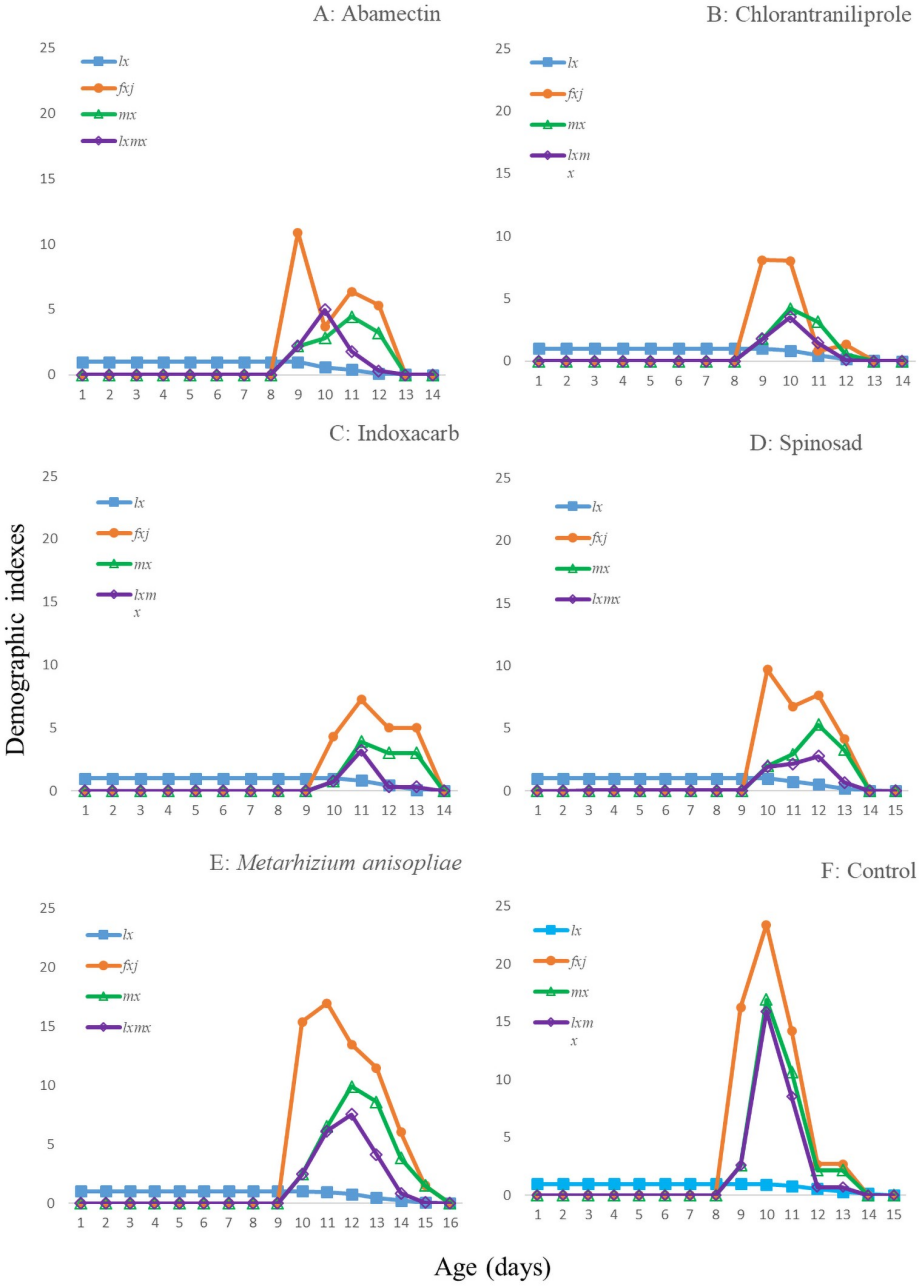
Age-specific survival rate (*l*_*xj*_), female age-specific fecundity (*f*_*xj*_), age-specific fecundity of the total population (*m*_*x*_) and age-specific maternity (*l*_*x*_*m*_*x*_) of the *T*. *brassicae* exposed to different pesticides 48 hours after spraying, A: Abamectin, B: Chlorantraniliprole, C: Indoxacarb, D: Spinosad, E: *M*. *anisopliae*, F: control.

**Fig 3:**
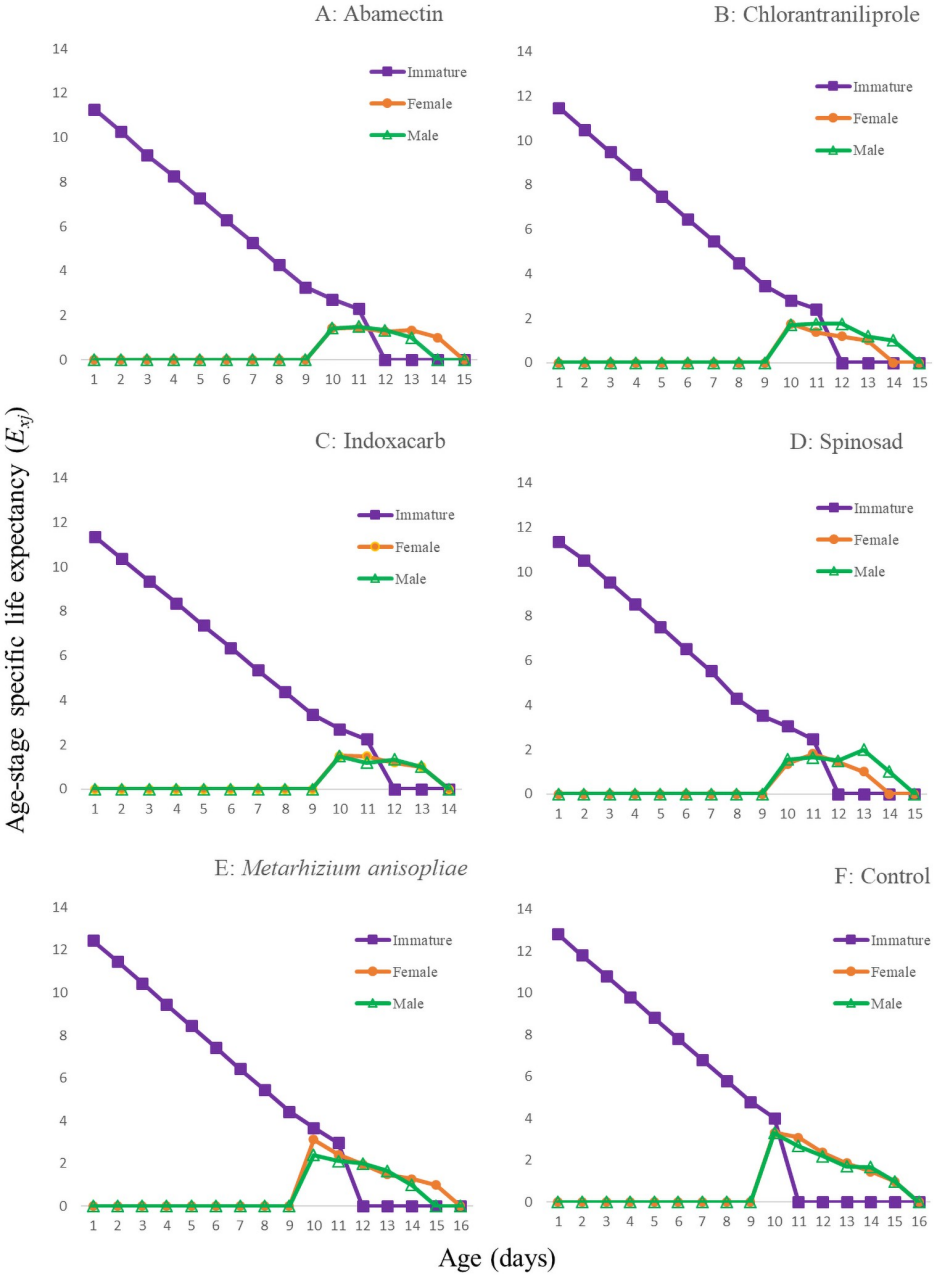
Age-stage specific life expectancy (*E*_*xj*_) of the *T*. *brassicae* exposed to different pesticides 48 hours after spraying, A: Abamectin, B: Chlorantraniliprole, C: Indoxacarb, D: Spinosad, E: *M*. *anisopliae*, F: control.

**Fig 4:**
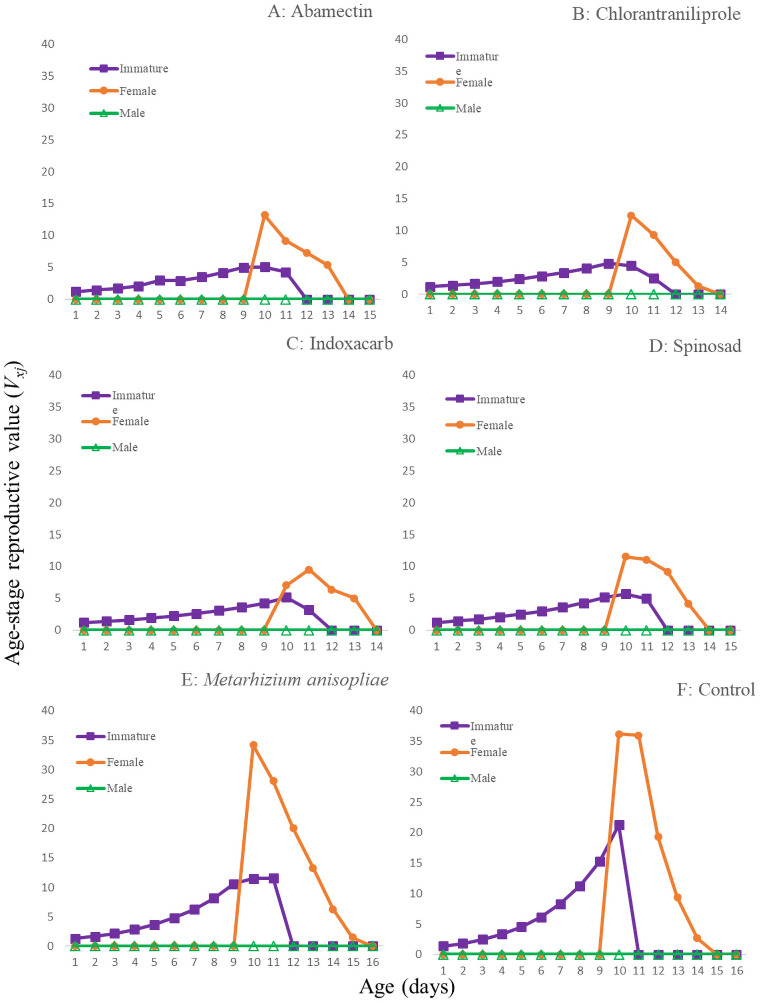
Age-stage reproductive value (*V*_*xj*_) of the *T*. *brassicae* exposed to different pesticides 48 hours after spraying, A: Abamectin, B: Chlorantraniliprole, C: Indoxacarb, D: Spinosad, E: *M*. *anisopliae*, F: control.

The age-stage survival rates (*S*_*xj*_*)* are shown in Fig 1. In all of the treatments, adults of both sexes emerged at day 8 or 9. The curves showed that a newly born eggs can survive until age *x* and develop to stage *j*. The female and male curves were different for all of the treatments. The population age-specific survival rate (*l*_*x*_), age-specific fecundity of the total population (*m*_*x*_), the age-specific maternity (*l*_*x*_*m*_*x*_) and fecundity (*F*_*x*_) are given in Fig 2. In addition, the first peak of *F*_*xj*_ were at 9 or 10d in the treatments. For *l*_*x*_*m*_*x*,_ these peaks occurred at 10-12d. The *E*_*xj*_ curves (Fig 3) show that the individuals of *T*. *brassicae* tend to live longer in control and in the *M*. *anisopliae* treatments than in other treatments. The maximum *V*_*xj*_ value was observed in the control and the *M*. *anisopliae* treatments (Fig 4). Only the curves at 48h are shown to enable a true comparison.

Although the insecticides and the entomopathogen had some negative effects on the parasitoid, a combination of them could increase the mortality rate of the *T*. *absoluta* eggs. According to the results in Table 3, the combination of *M*. *anisopliae* and *T*. *brassicae* had a synergistic effect when simultaneously applied. Other combinations showed additional effects. The longer developmental time of the infected host may extend the time available for parasitism, and thus a combination of these biological agents, particularly in simultaneous application, may be more effective than the sole use of insecticides on the parasitoid.

**Table 3:** The mortality rates of *Tuta absoluta* in an integrated system including the parasitoid *Trichogramma brassicae* + the entomopathogenic fungus, *Metarhizium anisopliae*, or the chemical insecticides spinosad, abamectin, indoxacarb and chlorantraniliprole.

Combination	Timing (h) of parasitoid release after the treatment	% observed mortality	% expected mortality	χ^2^(df)	Type of effect
**Spinosad**	**0**	**74.47**	**71.11**	**0.16 (1)**	**additive**
	**24**	**77.54**	**68.84**	**1.10 (1)**	**additive**
	**48**	**78.39**	**68.18**	**1.53 (1)**	**additive**
**Abamectin**	**0**	**68.82**	**74.15**	**0.38 (1)**	**additive**
	**24**	**70.52**	**70.48**	**0.28 (1)**	**additive**
	**48**	**74.53**	**67.32**	**0.77 (1)**	**additive**
**Indoxacarb**	**0**	**70.46**	**69.59**	**0.01 (1)**	**additive**
	**24**	**72.03**	**68.02**	**0.24 (1)**	**additive**
	**48**	**71.68**	**69.90**	**0.04 (1)**	**additive**
**Chlorantraniliprole**	**0**	**73.25**	**73.39**	**0.0003 (1)**	**additive**
	**24**	**74.10**	**68.84**	**0.40 (1)**	**additive**
	**48**	**74.84**	**67.32**	**0.84 (1)**	**additive**
***Metarhizium anisopliae***	**0**	**94.30**	**76.44**	**4.17 (1)**	**synergistic**
	**24**	**94.30**	**84.00**	**1.26 (1)**	**additive**
	**48**	**95.00**	**84.00**	**1.44 (1)**	**additive**

## Discussion

Biological control agents are often more sensitive to insecticides than targeted pests, possibly due to the shorter exposure time to the insecticides and the lower doses they receive [89]. The physiological selectivity mechanism of spinosad is unknown, so researchers cannot explain why resistance to spinosad has evolved in insects. However, a low penetration rate into the integument, a change in the site of activity or an increased insecticide metabolism rate are possible explanations for spinosad selectivity in wasps. Fernandes et al. [90] suggested that spinosad can be used as a moderately toxic compound for Vespidae and Apidae. They explained that the low penetration rate is due to cohesion with the integument and the large molecular weight of spinosad. In our study, no selectivity was observed between the parasitoid *T*. *brassicae* and its host *T*. *absoluta*. Suh et al. [91] also evaluated spinosad as a toxic compound for *Trichogramma exiguum*. Parsaeyan et al. [92] found that spinosad was the most toxic insecticide for *T*. *brassicae* whereas chlorantraniliprole was a safe compound, and that the insecticide residue changed the life table parameters of *T*. *brassicae*. However, Hewa-Kapuge et al. [93] reported that indoxacarb was a low toxicant compound for *Trichogramma* nr. *brassicae* in laboratory conditions and reduced adult emergence in the field. They also reported that emamectin was a moderately toxic insecticide against parasitoids. In the current study, abamectin and indoxacarb were slightly and moderately toxic for *T*. *brassicae*. The slight difference observed between our study and that of Hewa-Kapuge et al. [93] may be partly due to the different eggshell characteristics of the different hosts used in these studies. They used *H*. *armigera*, which has a thicker eggshell, as the host.

In addition, low-level lethal effects and life table parameters can also determine the degree of toxicity of a pesticide on natural enemies and pests [94]. Lundgren and Heimpel [95] documented that the longevity of *T*. *brassicae* was four and two days with and without feeding on honey. Orr et al. [46] recorded longevity to be four days for *T*. *brassicae*. These studies partially support our findings that *T*. *brassicae* adults can live five to seven days. By contrast, Afshari et al. [96], showed that indoxacarb reduced the longevity and efficiency of *T*. *brassicae*. Suh et al. [91] also reported that spinosad reduced the emergence rate and longevity of *T*. *exiguum* on *Helicoverpa armigera* (Boddie) eggs. Spinosad did not change the fecundity, sex ratio or frequency of brachyptery. Spinosad was classified as moderately toxic on *T*. *exiguum* females. Thus, spinosad appeared unable to penetrate the host egg, and the parasitoid adults were only affected by spinosad at emergence. However, in our study, all chemical insecticides (spinosad, abamectin, indoxacarb and chlorantraniliprole) resulted in changes to all of the life table parameters of the parasitoid. Differences in the parasitoid species and the insecticide doses may partly explain differences in our results. The size and shell thickness of the host egg can also alter the fecundity of *Trichogramma* spp [97].

Conversely, Medina et al. [98] showed that the first and second larval stages of *Hyposoter didymator* (Thunberg) (Hym., Ichneumonidae) were less affected than the third instar larvae within the body of the host, for larva of *Spodoptera littoralis* (Boisduval) (Lep.: Noctuidae). As the 1^st^ and the 2^nd^ instar larvae feed on the host hemolymph, they ingest less spinosad residues than the 3^rd^ instar larvae, which feed on the cellular tissue. In addition, adults are affected more through oral rather than contact exposure. The adults chew the silken cocoon and thus intake spinosad residue and then die. Spinosad was reported to have higher oral toxicity proportional to contact toxicity for this parasitoid, because the thick cuticle of the host acts as a preventive barrier. The undesirable effects of spinosad on *T*. *brassicae* life table parameters may be due to the aforementioned reasons. Whether the contact or the oral effects of spinosad are stronger depend on both the host and the natural enemy species. Ruiz et al. [99] indicated that the contact effect of spinosad is greater than the oral effect for *Diachasmimorpha longicaudata* (Ashmead) (Hym.: Braconidae). They also found that low lethal doses of spinosad had deleterious effects on fecundity, survival and partly on sex ratio.

Fernandes et al. [100] documented that the reduction in fecundity and the size of the parasitoid progeny may be due to the accumulation of spinosad in the ovaries. This can explain the reduction of fecundity found in our study. Similarly, Schneider et al. [101] reported lethal and low lethal effects of spinosad on *H*. *didymator* (Thunberg). However, the mechanism of spinosad toxicity on parasitoid wasps is relatively unknown. The parasitoids intake so few host egg chorion that no detectable effects may be observed. The spinosad that penetrates into the eggs is possibly responsible for the effects. Cônsoli et al. [102] reported that this insecticide on *Trichogramma galloi* Zucchi affected the neural system and particularly the nicotinic receptors that cause parasitoid paralysis. The female parasitoids are also directly exposed to insecticides during egg probing and host feeding. Spinosad had a deleterious effect on females on the first day. All of these theories can explain the low lethal effects of spinosad. Hossain and Poehling [103] showed that a part of the insecticide penetrates the underside layers of host eggs and larval skin and the remainder is absorbed by the host tissue and is fed by the parasitoid. Thus, the parasitoid is exposed to low lethal doses of abamectin and spinosad. However, Blibech et al. [104] documented that spinosad was a safer compound for three species of *Trichogramma* when compared to deltamethrin. This is in contrast to our study, as we categorize spinosad as similar to the other insecticides. They found spinosad to be a low to moderate toxic compound with these parasitoids and argued that there is a difference among species in terms of their response to different insecticides.

Abamectin not only has a moderate lethal effect on *T*. *absoluta* [72], but also causes low lethal effects on biological control agents such as *T*. *brassicae*, and thus it should be used cautiously [69]. Undesirable sublethal effects of both abamectin and Spinosad, such as reduced fecundity and longevity, were reported for *Bracon nigrican*s Szépligeti (Hymenoptera: Braconidae). These results support ours in terms of the detectable sublethal effects on the life table parameters of the parasitoid, which in this case is *T*. *brassicae*. Hewa-Kapuge et al. [93] evaluated emamectin as a very toxic compound for *Trichogramma* nr. *brassicae*, but found indoxacarb to be safe for this parasitoid under laboratory conditions. However, indoxacarb had a toxic effect on *T*. nr. *brassicae* in field experiments, which may be due to the high temperatures in the field. Wang et al. [105] observed adverse effects of abamectin on *T*. *nubilale* as did Ertle and Davis in their experiment. Cônsoli et al. [106] categorized the abamectin as moderately toxic for *T*. *pretiosum* as it reduced adult emergence and parasitism. However, in our study we found abamectin to also be slightly toxic. This is possibly due to the effect of insecticides on oogenesis and the developmental stages of the parasitoid, which can affect the results. According to Carvalho et al. [107], a residue of abamectin or spinosad on host egg chorion may cause adult mortality or reduce longevity and fecundity in *T*. *pretiosum*.

As high amounts of compounds can be transferred from the hemolymph to ovaries, spinosad or other insecticides may be traced in eggs and cause deleterious effects on the next generation [98]. Medina et al. [98] documented that a 55% level of spinosad appeared in the ovaries of *H*. *didymator* (Thunberg). Contrary to our findings, the results of Sattar et al. [108] suggested that spinosad and emamectin benzoate were very toxic compounds for *T*. *chiloni*s. In their study, indoxacarb reduced fecundity and was slightly harmful, except for the egg. Low lethal doses of insecticides may eventually affect the behavior and physiological state of a parasitoid without a parallel increase in mortality, thus creating a new population equilibrium. We also found that indoxacarb had not only a moderate lethal effect, but also had deleterious sublethal effects like the other tested insecticides. Delpuech et al. [109, 110] reported a weakening response of males to females after insecticide treatments. Consequently, fecundity was reduced and the sex ratio became male-biased.

Although specific insecticides had adverse effects on parasitoids, the combination of them was additive and synergic. Ashraf Khan [111] also documented that the residual of insecticides such as abamectin can reduce the emergence and parasitism of *T*. *chilonis*, but they can be integrated for parasitoids in pest management. However, Blibech et al. [104] disagreed with these results, and revealed that the use of deltamethrin and spinosad with *Trichogramma oleae*, *T*. *cacoeciae* and *T*. *bourarachae* was effective in the integrated pest management of olive tree ecosystems.

A major task in IPM research studies is to develop pest control strategies that better exploit biological control services and reduce chemical inputs. Better knowledge of chemical insecticides, biological mortality factors and combining synergistic control measures can help obtain promising results. Based on the results of this study, it appears that a combination of insecticides for a parasitoid can reduce pest damage. We also found the entomopatogen fungus to be safe in low lethal doses, and *M*. *anisopliae* was then a more compatible compound for the parasitoid compared with chemical insecticides. The entomopathogen fungus was not only safer for the parasitoid but also showed a synergistic effect on leaf miner egg mortality in combination. Nevertheless, more experiments are required and our results need validation through field studies, to gain further insights into *Trichogramma* species effectiveness in integrated control programs.

## Supporting information

S1 File (XLSX)
